# Intrabiofidelity: A Methodological Proposal to Simulate the Internal Trabecular Structure of Bone Tissue in Finite Element Biomechanical Models

**DOI:** 10.3390/bioengineering13070797

**Published:** 2026-07-12

**Authors:** Rodrigo Arturo Marquet-Rivera, Jesús Alejandro Serrato-Pedrosa, Verónica Loera-Castañeda, Juan Alejandro Vázquez Feijoo, Octavio Alejandro Mastache-Miranda, Rosa Alicia Hernández-Vázquez

**Affiliations:** 1 Escuela Superior de Comercio y Administración Unidad Tepepan, Instituto Politécnico Nacional, Anillo Periférico Sur Manuel Gómez Morín 4863, Colonia Ampliación Tepepan, Alcaldía Coyoacán, Ciudad de México 16020, Mexico; rmarquetr@ipn.mx; 2 Escuela Superior de Ingeniería Mecánica y Eléctrica Unidad Culhuacán, Instituto Politécnico Nacional, Av. Sta. Ana 1000, San Francisco Culhuacán, Colonia Culhuacán CTM V, Alcaldía Coyoacán, Ciudad de México 04440, Mexico; 3 CIIDIR-Durango, Instituto Politécnico Nacional, Calle Sigma 119, Fraccionamiento 20 de Noviembre II, Durango 34220, Mexico; alejandroserrato@live.com.mx (J.A.S.-P.); vloera@ipn.mx (V.L.-C.); 4 Sección de Estudios de Posgrado e Investigación, Escuela Superior de Ingeniería Mecánica y Eléctrica Unidad Zacatenco, Instituto Politécnico Nacional, Edificio 5, 2do piso, Lindavista, Ciudad de México 07738, Mexico; 5 Unidad Profesional Interdisciplinaria de Energía y Movilidad, Instituto Politécnico Nacional, Av. Wilfrido Massieu, Adolfo López Mateos S/N, Nueva Industrial Vallejo, Gustavo A. Madero, Ciudad de México 07738, Mexico; omastachem@ipn.mx; 6 División de Mecatrónica, Universidad Politécnica del Valle de México, Av. Mexiquense s/n esquina Av. Universidad Politécnica, Col. Villa Esmeralda, Tultitlán 54910, Mexico

**Keywords:** biomodel, biofidelity, intrabiofidelity, finite element method, trabecular bone, MRI-based modeling, computational biomechanics

## Abstract

Computational biomechanics has grown substantially alongside imaging modalities such as computed tomography (CT) and magnetic resonance imaging (MRI), which together enable high-fidelity biomodels of both hard and soft tissues. Most such biomodels, however, are represented as continuous homogeneous solids, limiting their capacity to reproduce the internal architecture of living tissues. Micro-finite element (μFE) analysis has addressed this limitation for bone at sub-millimetric scales using micro-CT data, but its adoption remains constrained by scanner availability, computational cost, and workflow complexity. This work proposes a methodological framework, termed *intrabiofidelity*, as a taxonomic descriptor complementary to *biofidelity* that characterizes the degree to which a biomodel reproduces the internal morphology and morphometry of a tissue. A reproducible pipeline based on ScanIP^®^ segmentation of MRI-derived DICOM data, SolidWorks^®^ solidification, and ANSYS^®^ Workbench finite element analysis is presented, through which a macro-scale trabecular representation is extracted from the distal femoral cancellous bone and integrated into a knee biomodel. Two numerical analyses were performed under an equivalent bipodal-standing load with orthotropic material properties for cortical and trabecular bone: one with external biofidelity only (Case 1), and one incorporating macro-scale intrabiofidelity in the trabecular bone (Case 2). The introduction of intrabiofidelity produced a substantial redistribution of peak von Mises stress between compartments. Trabecular peak stress increased from 2.66 to 12.10 MPa (a 4.5-fold elevation), while cortical peak stress decreased from 56.25 to 45.97 MPa (an 18.3% reduction), whereas the volume-averaged stress remained essentially unchanged in both tissues, indicating that intrabiofidelity primarily affects local concentrations rather than the bulk stress state. Principal stress data further revealed that the trabecular region transitions from a low-stress, predominantly compressive state in Case 1 to one in which substantial local tensile and compressive concentrations of comparable magnitude coexist in Case 2. The proposed methodology provides an accessible workflow for macro-scale integration of internal bone architecture using routinely available MRI data and commercial FEA software, and introduces *intrabiofidelity* as a terminological complement useful for teaching and for systematically documenting the fidelity of computational biomodels.

## 1. Introduction

Finite element analysis (FEA) has become a standard tool in computational biomechanics for studying the mechanical behavior of biological tissues and the interaction between tissues and orthopedic devices [1,2,3,4]. The methodological pipeline supporting most modern FEA of musculoskeletal structures is well established: three-dimensional imaging through computed tomography (CT) or magnetic resonance imaging (MRI) produces stacks of Digital Imaging and Communications in Medicine (DICOM) slices, which are subsequently segmented, meshed, and exported as solid geometries to commercial finite element solvers [1,5]. The three-dimensional models obtained through this pipeline are commonly referred to as *biomodels* [6,7].

Within this pipeline, the concept of *biofidelity* is routinely invoked (though rarely defined operationally) to describe the degree to which a biomodel reproduces the morphology and morphometry of the real tissue. In practice, biofidelity in the FEA literature almost always refers to the *external* surface of a structure: the outer contour of a bone, the shape of a joint, the silhouette of an organ. Substantial methodological effort has been devoted to improving external biofidelity through refined segmentation, surface smoothing, and mesh quality control [8,9,10,11,12]. The internal architecture of the tissue, by contrast, is typically treated as a continuous homogeneous solid, even when the real tissue is known to exhibit porosities, fibers, trabeculae, or ducts that influence its mechanical behavior.

This external/internal distinction is not new in bone biomechanics. Since the mid-1990s, micro-finite element analysis (μFE) has provided a rigorous framework for the explicit representation of trabecular architecture in FEA. Seminal work by Van Rietbergen and colleagues established voxel-based μFE as a method to convert high-resolution micro-computed tomography (μCT) images of cancellous bone into finite element meshes that preserve the native trabecular network [13,14]. Rüegsegger, Müller, and colleagues subsequently developed μCT systems capable of non-destructive in vitro characterization of bone architecture at resolutions of tens of micrometers [15], and the μFE methodology has since been refined and validated against experimental measurements across a broad range of anatomical sites and loading scenarios [16,17,18,19]. Comparisons between micro-level and continuum-level voxel models have also clarified the conditions under which homogenized representations remain accurate at the apparent scale [17,18]. More recently, Pahr, Zysset, and coworkers have systematically characterized the relationship between trabecular morphometry (quantified through indices such as bone volume fraction (BV/TV), trabecular thickness (Tb.Th), trabecular separation (Tb.Sp), and anisotropy) and the apparent mechanical properties predicted by μFE, and have formalized homogenized-FE (hFE) approaches that encode this information into coarser, computationally efficient models [20,21,22,23]. Together, the μFE and hFE families of methods constitute the state of the art for explicit or implicit representation of trabecular architecture in FEA of bone.

μFE and hFE, however, have practical requirements that constrain their use outside specialized research centers. μFE depends on high-resolution μCT, which is typically limited to excised specimens and small in vivo peripheral sites; the resulting meshes routinely contain tens to hundreds of millions of elements and require high-performance computing resources for solution [13,24]. hFE alleviates the computational demand but still relies on μCT-derived morphometric indices to parameterize the apparent properties of the continuum. For research and educational contexts in which μCT is unavailable (including most clinical biomechanics laboratories and many applied-engineering groups), the practical alternative has been to use CT- or MRI-based biomodels in which trabecular bone is represented as a continuous isotropic or orthotropic solid, with no explicit internal architecture. This is a pragmatic simplification, but it introduces an asymmetry in how biomodels are documented and discussed: a model that reproduces the external shape of a bone with high fidelity while representing its interior as a continuous solid is routinely described as *high-fidelity*, without any systematic qualification of what *fidelity* means at the internal scale.

Although μCT-based μFE has extensively characterized trabecular bone mechanics at the sub-millimetric scale, the FEA literature on applied orthopedic biomechanics (covering prosthesis analysis, fixation devices, joint injury, and preliminary implant design) relies almost universally on biomodels in which trabecular bone is represented as a continuous solid with apparent material properties, without explicit internal geometry. This asymmetry reflects practical constraints rather than methodological preference: research groups working with anatomically complete biomodels rarely have μCT access to the segment of interest, and the systematic integration of macro-scale trabecular geometry into clinical biomodels has not been established as standard practice. The intrabiofidelity framework proposed here addresses precisely this gap.

The present work addresses this terminological and methodological asymmetry in two ways. First, we propose the term *intrabiofidelity* (from Latin *intra-*, inside) as a taxonomic descriptor complementary to *biofidelity*. Whereas biofidelity denotes the morphological and morphometric reproduction of the *external* surface of a tissue, intrabiofidelity denotes that of its *internal* structure at a specified scale. Introducing this term is not intended to claim novelty in the physical modeling of trabecular architecture (a problem already addressed by μFE). Under this vocabulary, μFE models of trabecular bone have, by construction, very high intrabiofidelity at the sub-millimetric scale; hFE models translate intrabiofidelity into continuum-level apparent properties; and conventional CT/MRI-based biomodels of bone have, in most cases, low intrabiofidelity regardless of how high their external biofidelity may be.

Second, we describe a pragmatic, macro-scale pipeline through which internal bone architecture can be partially incorporated into FEA biomodels using routinely available imaging (MRI) and standard commercial software (ScanIP^®^, SolidWorks^®^, ANSYS^®^ Workbench). The methodology is intended as an intermediate step between a fully continuous biomodel and a full μFE representation: it does not resolve individual trabeculae at native scale, but introduces explicit internal discontinuities extracted from the segmented trabecular volume, with the aim of representing the presence of internal porosity at a scale compatible with MRI resolution. To illustrate the approach, a biomodel of the distal femur is constructed with orthotropic material properties for cortical and trabecular bone consistent with values previously reported and used by our group in FEA studies [6,25], and two numerical analyses under an equivalent bipodal-standing load are compared: one with external biofidelity only, and one combining biofidelity with macro-scale intrabiofidelity.

The objective of this work is therefore twofold: (i) to introduce and operationally define *intrabiofidelity* as a complementary descriptor of biomodel quality, situating it explicitly in relation to the μFE and hFE literature; and (ii) to present and illustrate a reproducible MRI-based pipeline for macro-scale integration of internal trabecular architecture into FEA biomodels using commercial software. The contribution is methodological and terminological; it does not claim to improve upon the predictive accuracy of μFE, nor to provide a validated alternative to established homogenization schemes. The scope and limitations of this framing are made explicit in Section 4.

## 2. Materials and Methods

**Figure 1:**
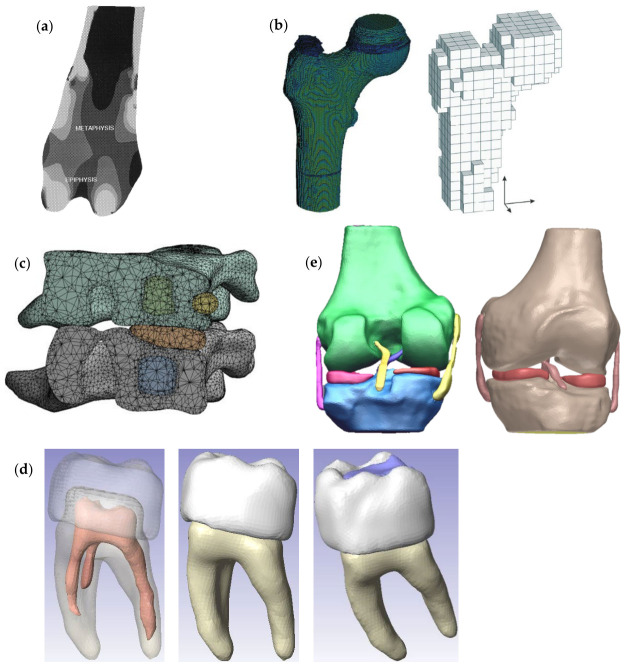
Evolution of biomodels: (**a**) 1997 model [26]; (**b**) 2012 model [27]; (**c**) 2016 model [28]; (**d**) 2020 model [7]; (**e**) 2021 model [6].

Computational modeling of living tissues is a non-invasive technique that has improved the design of prostheses and orthotic devices by allowing their analysis as engineering elements under physiological loading conditions. Over time, biomodel generation has evolved from regular geometric representations to complex anatomical reconstructions that better approximate real tissue morphology. Refining external geometry increases result accuracy but also demands higher computational resources, longer processing times, high-precision imaging, and deeper anatomical knowledge [11,12] (Figure 1).

**Figure 2:**
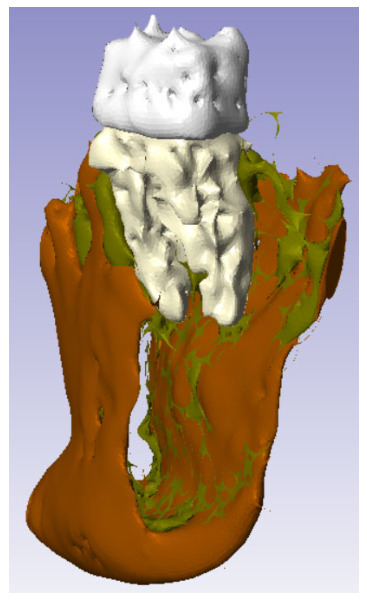
Typical problems in generating biomodels.

An additional consideration is that migration of geometries between different CAD/FEM software often introduces surface defects whose severity depends on biomodel quality and available computational resources. Typical defects include gaps (separations between faces), bad edges (triangular elements connected only by their borders), holes (regions where information is lost during export), and shells (internal faces that self-intersect and add spurious geometry) (Figure 2). These defects become particularly problematic when attempting to represent internal tissue architecture, because the discontinuities of porous regions such as trabecular bone amplify the frequency and impact of such errors. The present work, therefore, describes a procedure to mitigate these problems in the specific case of macro-scale trabecular representation, supporting the proposed concept of *intrabiofidelity*.

### 2.1. Biomodel Generation from MRI

Several methodologies have been reported for the generation of computational biomodels of living tissues [6,9,11,29]. In the present work, biomodels are generated from MRI studies, from which DICOM files are obtained. DICOM files contain a sequence of images or sections of the biological tissue that, when stacked, form the three-dimensional element for visualization. To manipulate these files, the ScanIP^®^ computer program is used; within this program, the point cloud used for subsequent solidification is generated.

Within ScanIP^®^, the DICOM file is imported, and the region of interest is delimited to reduce the computational cost of point cloud generation. Once histogram parameters are defined, four simultaneous views are displayed: sagittal, transverse, and frontal planes, plus a three-dimensional preview of the point cloud. Starting from a selected slice, the pixels corresponding to the tissue of interest are painted; when these pixels are propagated across successive DICOM slices, voxels are formed, and their spatial grouping defines the morphology of each tissue. At this stage, the resulting three-dimensional element is still a point cloud and not yet a surface or solid body.

For the present study, four tissues were segmented in ScanIP^®^: cortical bone and trabecular bone of the distal femur as hard tissues, and internal and external menisci as soft tissues. This yields four biomodels with high external biofidelity, in the sense that the external morphology and morphometry closely match the imaged anatomy. Living tissues, however, exhibit behavior that is also governed by internal irregularities in the case of trabecular bone, a network of trabeculae with variable thickness, orientation, pronounced or rounded vertices, and spatial randomness. Reproducing these features at a macro-scale motivates the generation of a fifth biomodel, explicitly representing the trabeculae extracted from the cancellous bone. This internal representation is what is termed here intrabiofidelity: the characteristic of morphologically and morphometrically reproducing, at a given resolution, the internal structure of a tissue, as a descriptor complementary to biofidelity [13,20].

### 2.2. Extraction of the Trabecular Representation

The fifth biomodel (representing the trabeculae) is generated by subtracting internal voids from the solid point cloud of the trabecular bone. Because erasing voxels in the DICOM slices generates geometric inconsistencies in the point cloud (open faces, bad edges, and intersecting shells), the process is performed stepwise, progressing through the distal femur in cranio-caudal direction. For each slice, internal voids are defined conservatively to preserve connectivity, and the remaining point cloud is verified before moving to the next slice.

The first tissue simulated is the cortical bone of the distal third of the femur, followed by the trabecular bone, the internal and external menisci, and finally the trabecular representation obtained by extraction. The resulting point clouds are then exported to SolidWorks 2025^®^ for solidification, where the trabecular representation is likewise extracted. Solidified biomodels are subsequently imported into ANSYS^®^ Workbench for discretization and numerical analysis.

### 2.3. FEA Configuration

Two numerical analyses were performed in ANSYS^®^ Workbench. In the first analysis (Case 1), the trabecular bone was modeled as a continuous solid with no internal discontinuities; the biomodel therefore presents external biofidelity only. In the second analysis (Case 2), the same boundary conditions, loads, and material properties were applied, but the trabecular bone incorporated the extracted trabecular representation, introducing macro-scale intrabiofidelity. The element used for discretization was the 20-node tetrahedral solid SOLID187. Mesh quality was evaluated using the skewness metric [30]. Detailed mesh statistics and a discussion of discretization considerations specific to the intrabiofidel geometry are reported in Section 2.7.

### 2.4. Boundary Conditions and Loading

A 90 kg subject in a bipodal-standing position is considered. Under this condition, the body weight is distributed between both lower limbs, so each knee is subjected to approximately half the body weight (45 kg), corresponding to a magnitude of 441.45 N. This value represents a conservative quasi-static estimate of the external load applied to the knee in bipodal standing [31] and was used to enable a direct comparison between Case 1 and Case 2 under identical boundary conditions. The limitations of this simplification, particularly the omission of muscle contributions that substantially increase tibiofemoral contact forces during physiological activities [32,33,34], are acknowledged in Section 4.

Contact conditions were defined in accordance with the anatomical configuration of the model. Bonded contact was assigned between cortical and trabecular bone, assuming continuity of the osseous tissue. Between the cortical bone of the femur and the menisci, frictionless contact was defined as a first approximation of articular interaction. A comprehensive list of contact pairs is provided in Table 1.

### 2.5. Material Properties

Orthotropic linear-elastic material properties were assigned to cortical and trabecular bone. The orthotropic dataset adopted in the present work follows directly that established by Serrato-Pedrosa et al. [25] for FEA of bone tissue at the same anatomical scale, and is consistent with prior FEA studies of bone performed by our group [6]. Orthotropic rather than isotropic properties were used to better represent the directional mechanical behavior of bone, in which the response is strongly dependent on loading orientation relative to the trabecular architecture [35,36,37]. The trabecular Young’s moduli adopted here (Ex = 144, Ey = 99, Ez = 344 MPa) fall within the range of values reported by Wu et al. [34] for human trabecular bone at the tissue level, and the orthotropic structure of the elasticity tensor is consistent with the principal directions of the trabecular network at the distal femur [3]. Mechanical properties of the meniscus were taken from the established literature values for human knee menisci under static loading [38], with a Poisson ratio of 0.45 consistent with the near-incompressible behavior of hydrated soft fibrocartilaginous tissue [39]. All materials were assumed to be linear-elastic, continuous, and homogeneous at the macro-continuum level; the non-homogeneous nature of trabecular bone was accounted for geometrically through the intrabiofidelity extraction described in Section 2.1 and Section 2.2. Table 2 summarizes the mechanical properties used in this study.

Note on the cortical Poisson ratio: the cortical Poisson ratio, νxy = 0.55, reported in Table 2, follows the orthotropic dataset adopted in our previous FEA studies [6,25]. Poisson ratios above 0.5 have been reported in highly anisotropic biological tissues and in orthotropic formulations of cortical bone, and do not violate thermodynamic stability provided the full compliance tensor remains positive-definite [35,37]. We retain the value used in our previous work for consistency and traceability with Serrato-Pedrosa et al. [25], acknowledging that alternative isotropic formulations typically assume ν ≈ 0.3.

### 2.6. Discretization and Intrabiofidelity-Related Mesh Considerations

All biomodels were discretized with SOLID187 elements. The nominal element size was controlled globally and locally refined in the regions surrounding the trabecular representation to preserve geometric detail. Because the inclusion of intrabiofidelity introduces numerous internal discontinuities, mesh quality was closely monitored: regions of high skewness tend to concentrate around thin trabecular features and could otherwise compromise solution accuracy. The skewness metric [30] was used to quantify element distortion. Total numbers of elements and nodes per case, together with a detailed discussion of the quality of the intrabiofidelity-related mesh, are reported in Section 2.7.

### 2.7. Mesh Quality and Discretization Considerations

The discretized biomodel corresponding to Case 2 (with intrabiofidelity) comprises 2,987,600 nodes and 1,831,736 elements (Table 3), with a geometry bounding-box diagonal of 194.29 mm. Mesh quality was assessed using the skewness metric [30]. The average skewness of the Case 2 mesh was 0.45, placing the global mesh quality in the “good” range according to ANSYS Workbench mesh-metric conventions. The maximum skewness reached 1.000, indicating the presence of isolated degenerate elements in the mesh.

The presence of degenerate elements at the upper tail of the skewness distribution is a direct consequence of the macro-scale trabecular geometry obtained from MRI-derived DICOM segmentation. Thin trabecular features and the intersections between trabeculae generate sub-millimetric surface artifacts with effective edge lengths of the order of 10^−5^ mm, which cannot be meshed with well-conditioned tetrahedral elements. Several strategies were applied to mitigate this effect prior to meshing: (i) adaptive Defeature Size was configured on the mesh object, (ii) automatic Virtual Topology was inserted with “Low” behavior to merge small faces and collapse short edges, and (iii) increased Virtual Topology tolerance (“Medium” behavior) was subsequently attempted. None of these strategies eliminated the degenerate elements in the trabecular region without simultaneously removing anatomically meaningful trabecular features, and further refinement of the global element size resulted in meshing failures.

The reported mesh, therefore, represents the highest-quality discretization attainable for the present trabecular representation using the standard ANSYS Workbench meshing pipeline, and a conventional h-refinement convergence study in the strict sense could not be completed. The localized nature of the skewness outliers, together with the smooth and continuous displacement fields reported in Section 3, supports the interpretation that the global solution is not dominated by these outlier elements. This is discussed further as a methodological limitation in Section 4.3.

**Figure 3:**
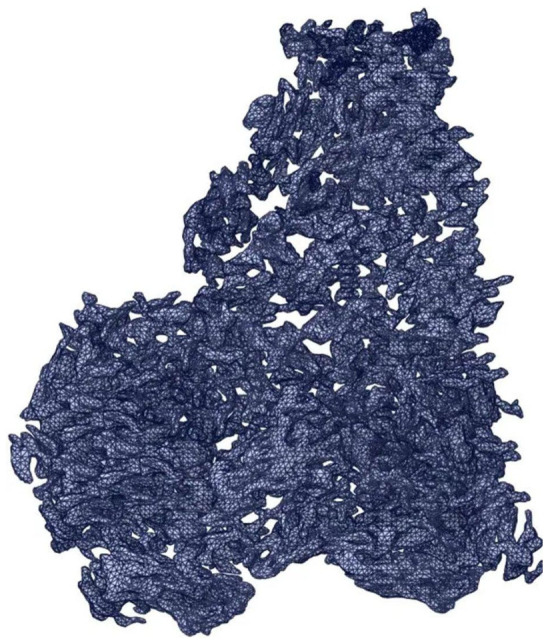
Tetrahedral discretization of the extracted trabecular biomodel used in Case 2 (intrabiofidelity). The image illustrates the geometric complexity of the macro-scale trabecular representation obtained through MRI segmentation and subsequent solidification: individual trabeculae, internal porosities, and trabecular junctions are all explicitly meshed with SOLID187 elements. The local mesh density adapts to the thickness of each trabecular feature, with finer elements near the narrowest cross-sections (“trabecular necks”) where stress concentrations develop in Case 2. The complexity visible here is what motivates the use of skewness-based mesh quality control (Table 3) and the discussion of meshing limitations in Section 2.7.

Case 1 (external biofidelity only) uses an equivalent biomodel with the trabecular bone represented as a continuous solid and was discretized with the same nominal element size. Because the trabecular region of Case 1 lacks internal surface detail, its mesh does not exhibit the degenerate elements observed in Case 2. The total numbers of elements and nodes for Case 1 are reported in Table 3 alongside Case 2 for direct comparison (Figure 3).

Peak values of total deformation, von Mises stress, and principal stresses for Case 1 and Case 2 are summarized in Table 4.

### 2.8. Volume Control Analyses

To address the methodological concern that the stress redistribution observed in Case 2 could be attributable to the volume reduction inherent to the explicit trabecular representation rather than to the architecture itself, two additional control analyses were performed, namely a material control (Case 3A) and a geometric control (Case 3C). Both controls used identical boundary conditions, loading conditions, and meshing strategy as Cases 1 and 2.

#### 2.8.1. Case 3A—Material Control (Apparent Density Scaling)

Case 3A retained the continuous solid geometry of Case 1 unchanged. The orthotropic elastic moduli of the trabecular compartment were scaled linearly by the volume fraction (factor = 0.873, corresponding to the 12.7% volume reduction observed in Case 2), following apparent density theory for trabecular bone [40,41]. Specifically, the Young’s moduli were reduced to Ex = 125.71 MPa, Ey = 86.43 MPa, and Ez = 300.31 MPa; the shear moduli to Gxy = 46.27 MPa, Gyz = 55.00 MPa, and Gxz = 39.29 MPa. Poisson ratios (νxy = 0.23, νyz = 0.11, νxz = 0.13) were not scaled, as they are dimensionless and not derived from material volume. This case isolates the effect of reduced material stiffness from any geometric or architectural effect.

#### 2.8.2. Case 3C—Geometric Control (Spherical Cavities)

Case 3C introduced a controlled geometric porosity into the continuous trabecular volume of Case 1 by Boolean subtraction of spherical cavities of uniform diameter (d = 7.833 mm). The number of cavities was calibrated such that the total volume removed approximated the 12.7% target of Case 2; the final geometric configuration removed 13.80% of the original trabecular volume (V_Case1 = 162,513.09 mm^3^ → V_Case3C = 140,085.94 mm^3^), a slight excess that makes this control conservative with respect to the hypothesis under test. The material properties used were identical to those of Case 1 (unmodified orthotropic constants from Table 2). This case isolates the effect of pure volume loss with regular (non-biofidel) geometric porosity from the effect of the biofidel trabecular architecture explicitly modeled in Case 2.

### 2.9. Quantitative Framework for Intrabiofidelity

To address the need for operational criteria associated with the concept of intrabiofidelity, a three-level classification framework is proposed, grounded in established bone morphometry indices (Bouxsein et al. [42]):

Level I (Low intrabiofidelity): Continuous solid representation of the trabecular compartment with orthotropic apparent-level properties. No internal architecture is geometrically resolved. The bone volume fraction (BV/TV) equals 1.0 by definition; trabecular thickness (Tb.Th) is not applicable. This level corresponds to Case 1 in the present study.

Level II (Intermediate intrabiofidelity): Partial representation of trabecular porosity through regular geometric primitives (e.g., spherical or cylindrical cavities) calibrated to match a target BV/TV value; architectural orientation and topology are not preserved. This level corresponds to Case 3C in the present study.

Level III (High intrabiofidelity): Explicit reconstruction of individual trabecular elements from medical imaging, with the achievable level depending on imaging resolution. In the present study, the use of clinical MRI (resolving features ≥ 363 µm as reported in as reported in Section 3.5) demonstrates that Level III is achievable for trabecular features above the resolution threshold; higher-resolution modalities such as HR-pQCT or µCT enable resolution of finer features but are not strictly required. The reconstruction preserves trabecular thickness (Tb.Th), spacing (Tb.Sp), number (Tb.N), and topological connectivity. BV/TV is measured directly from the reconstructed biomodel. This level corresponds to Case 2 in the present study, where BV/TV = 0.873 and mean Tb.Th = 363 ± 108 µm (as reported in Section 3.5).

This framework is proposed as a preliminary operationalization of intrabiofidelity, intended to facilitate reproducibility and inter-study comparison. Formal validation against µFE reference models is identified as a methodological priority for future work.

## 3. Results

Two numerical analyses were performed following the methodology described in Section 2. Case 1 corresponds to the high-biofidelity biomodel without intrabiofidelity, in which the trabecular bone is represented as a continuous, homogeneous solid with orthotropic material properties. Case 2 corresponds to the high-biofidelity biomodel with intrabiofidelity, in which the extracted trabecular representation was integrated into the trabecular bone. Both cases share identical material properties (Table 2), contacts (Table 1), boundary conditions, and external load. This section reports the results obtained for both cases: total deformation, von Mises equivalent stress (maximum and average), and principal stresses (maximum tensile and maximum compressive), as well as the morphometric characterization of the extracted trabecular representation.

Results are reported separately for cortical and trabecular bone, because the primary geometric difference between the two cases lies in the trabecular region, whose apparent mechanical behavior is expected to be most affected by the inclusion of intrabiofidelity. The cortical bone shares the same external geometry in both cases and is mechanically coupled to the trabecular region through bonded contact; any differences observed in the cortical output field between cases are therefore attributable to this coupling rather than to changes in cortical geometry itself.

### 3.1. Total Deformation Fields

**Figure 4:**
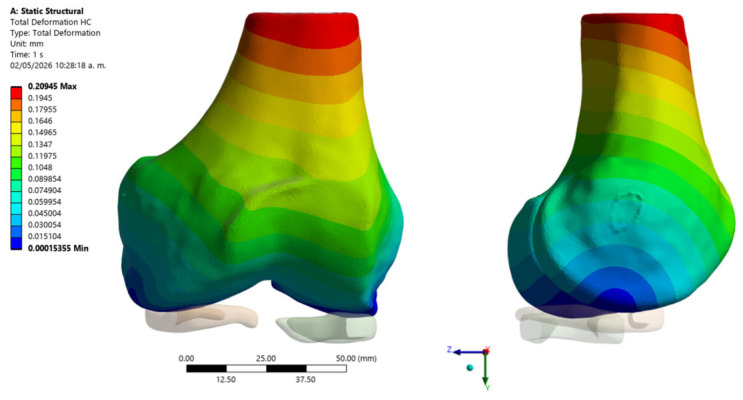
Total deformation field (mm) in cortical bone, Case 1 (without intrabiofidelity), under bipodal-standing load. The maximum value is 0.209 mm at the loading region. Isochromatic scale: 15 intervals, ANSYS Workbench output.

**Figure 5:**
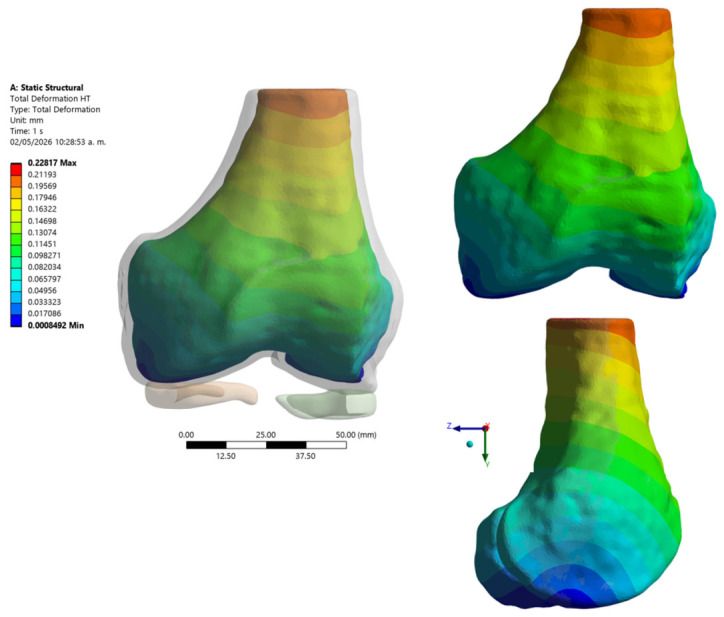
Total deformation field (mm) in trabecular bone, Case 1 (without intrabiofidelity), under bipodal-standing load. The maximum value is 0.228 mm at the loading region. The trabecular volume is here represented as a continuous solid; no internal porosity is present. Isochromatic scale: 15 intervals, ANSYS Workbench output.

Figure 4 and Figure 5 present the total deformation fields obtained in Case 1 (without intrabiofidelity) for cortical and trabecular bone, respectively. Peak cortical displacement was 0.209 mm, and peak trabecular displacement was 0.228 mm, both occurring in the loading region. The displacement fields are smooth and continuous, without internal discontinuities, since the trabecular region is represented as a continuous solid.

**Figure 6:**
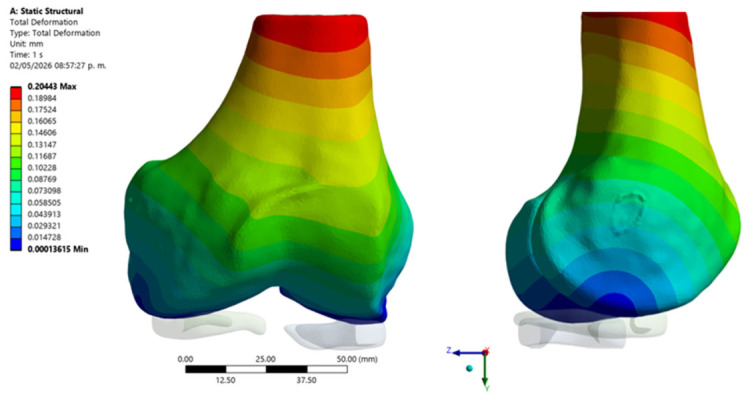
Total deformation field (mm) in cortical bone, Case 2 (with intrabiofidelity), under bipodal-standing load. The maximum value is 0.204 mm at the loading region. Compared to Case 1 (Figure 4), the peak displacement is reduced by 2.4%. Isochromatic scale: 15 intervals, ANSYS Workbench output.

**Figure 7:**
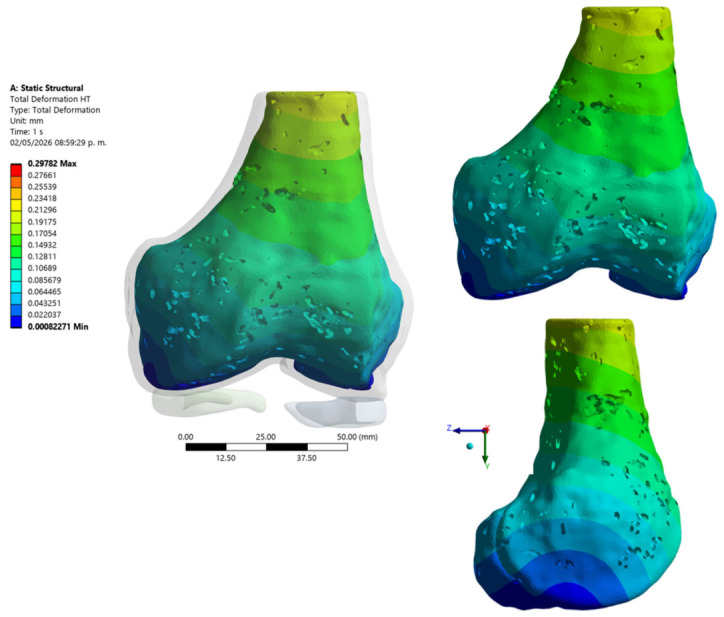
Total deformation field (mm) in trabecular bone, Case 2 (with intrabiofidelity), under bipodal-standing load. The maximum value is 0.298 mm at the loading region. The cavities visible on the trabecular volume reflect the internal porosity network extracted from the MRI-derived segmentation. Compared to Case 1 (Figure 5), the peak trabecular displacement is increased by 30.5%. Isochromatic scale: 15 intervals, ANSYS Workbench output.

Figure 6 and Figure 7 present the corresponding fields in Case 2 (with intrabiofidelity). Peak cortical displacement was 0.204 mm (a 2.4% reduction relative to Case 1), and peak trabecular displacement was 0.298 mm (a 30.5% increase). The cortical field of Case 2 closely tracks the underlying trabecular structure through the bonded contact at the cortical–trabecular interface (Section 2.4, Table 1), but with a peak value 31% lower than that of trabecular bone in this case (Table 4); the trabecular field, in turn, exhibits the small cavities characteristic of the extracted porosity network on the cross-section. The widened gap between cortical and trabecular peak displacements in Case 2, relative to Case 1, reflects the increased local compliance of the trabecular network at points where porosity reduces the effective load-bearing cross-section, while the cortical shell, partially relieved through load redistribution, exhibits slightly lower peak displacements than in Case 1.

### 3.2. Von Mises Stress Fields

**Figure 8:**
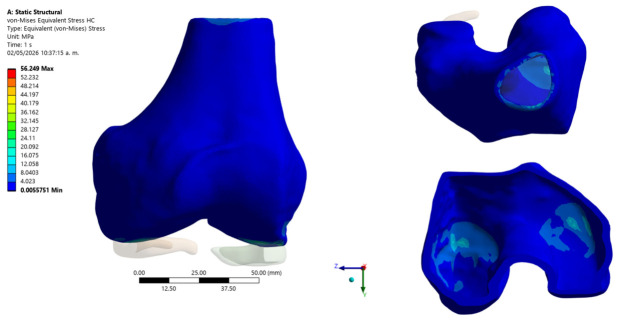
Von Mises equivalent stress field (MPa) in cortical bone, Case 1 (without intrabiofidelity), under bipodal-standing load. Maximum value 56.25 MPa at the loading region; volume-averaged value 1.28 MPa. The peak value remains well below the cortical yield strength of 115 MPa (Table 2), indicating elastic behavior. Isochromatic scale: 15 intervals, ANSYS Workbench output.

**Figure 9:**
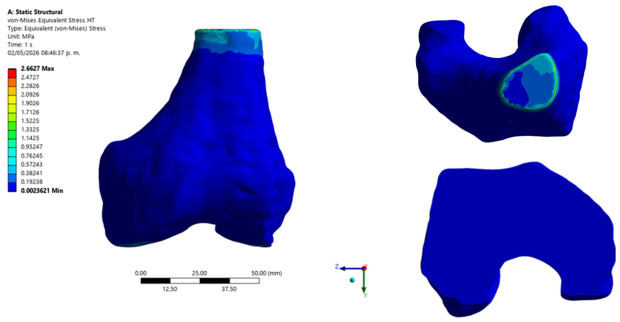
Von Mises equivalent stress field (MPa) in trabecular bone, Case 1 (without intrabiofidelity), under bipodal-standing load. Maximum value 2.66 MPa at the loading region; volume-averaged value 0.075 MPa. The trabecular continuum carries low stresses throughout, consistent with its lower stiffness and the predominance of cortical load transfer in this case. Isochromatic scale: 15 intervals, ANSYS Workbench output.

Figure 8 and Figure 9 present the von Mises equivalent stress distributions in Case 1 for cortical and trabecular bone, respectively. In cortical bone (Figure 8), peak von Mises stress was 56.25 MPa, localized in the loading region, with a volume-averaged value of 1.28 MPa. In trabecular bone (Figure 9), peak von Mises stress was 2.66 MPa, with a volume-averaged value of 0.075 MPa, indicating a low-stress bulk state of the trabecular continuum.

**Figure 10:**
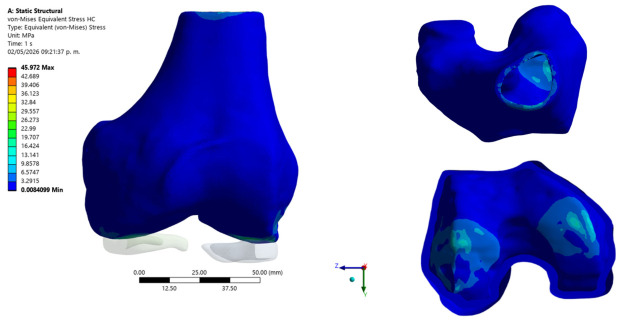
Von Mises equivalent stress field (MPa) in cortical bone, Case 2 (with intrabiofidelity), under bipodal-standing load. Maximum value 45.97 MPa at the loading region (an 18.3% reduction relative to Case 1, Figure 8); volume-averaged value 1.29 MPa (essentially unchanged from Case 1). Isochromatic scale: 15 intervals, ANSYS Workbench output.

**Figure 11:**
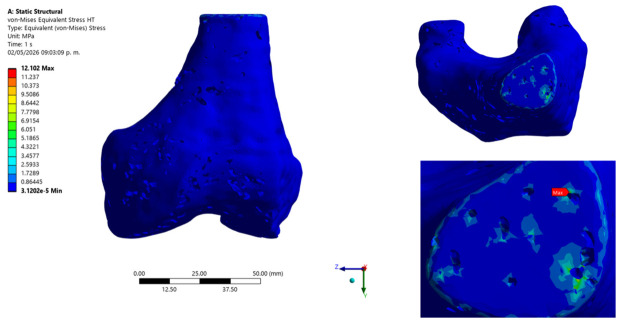
Cross-sectional detail of the von Mises equivalent stress field (MPa) in trabecular bone, Case 2 (with intrabiofidelity). The image shows a transverse cut through the trabecular volume, revealing the internal porosity network. The point of maximum stress (12.10 MPa) is localized at a narrow trabecular cross-section between extracted porosities; the surrounding trabecular volume carries stresses below 1 MPa (volume-averaged value: 0.086 MPa). This visual confirms the localized character of the stress concentrations introduced by intrabiofidelity. ANSYS Workbench output.

Figure 10 and Figure 11 present the corresponding fields in Case 2. In cortical bone (Figure 10), peak von Mises stress was 45.97 MPa, an 18.3% reduction relative to Case 1, with the volume-averaged value essentially unchanged (1.29 MPa). In trabecular bone (Figure 11), peak von Mises stress was 12.10 MPa, a 4.5-fold elevation relative to Case 1, while the volume-averaged value increased only modestly from 0.075 to 0.086 MPa (+15%). The peak stress is localized at trabecular features where porosity produces a stress concentration; Figure 11 shows a cross-sectional zoom of the trabecular volume in Case 2, illustrating how the local stress concentrations are confined to the narrow cross-sections between extracted porosities (“trabecular necks”), with the bulk of the surrounding trabecular volume carrying stresses below 1 MPa. Peak values for both tissues remain well below the corresponding yield strengths (Table 2), indicating elastic behavior throughout the analysis.

### 3.3. Quantitative Summary of Case 1 and Case 2

Table 4 summarizes the peak values of total deformation, von Mises stress (maximum and average), and principal stresses (maximum tensile and maximum compressive) obtained in Case 1 (external biofidelity only) and Case 2 (with macro-scale intrabiofidelity) for trabecular and cortical bone. Both cases share orthotropic material assignments for cortical and trabecular bone (Table 2), allowing direct evaluation of the effect of intrabiofidelity under identical loading and material conditions.

Total deformation: Maximum trabecular displacement was 0.228 mm in Case 1 and 0.298 mm in Case 2, corresponding to a relative increase of 30.5%. Maximum cortical displacement was 0.209 mm in Case 1 and 0.204 mm in Case 2, corresponding to a relative decrease of 2.4%. The displacement field of Case 2 thus exhibits a non-trivial asymmetry between tissues that is absent in Case 1: the trabecular region deforms substantially more than the cortical region in Case 2, while in Case 1, both tissues displace by comparable amounts. This pattern is consistent with the loss of effective stiffness in the trabecular region due to explicit internal porosity, with the cortical region partially relieved through the load-sharing mechanism described in Section 4.2.

Von Mises stress, maximum vs. average: In trabecular bone, peak von Mises stress increases from 2.66 MPa in Case 1 to 12.10 MPa in Case 2, a 4.5-fold elevation. The average von Mises stress over the trabecular volume, by contrast, increases only modestly, from 0.075 to 0.086 MPa (+15%). In cortical bone, peak von Mises stress decreases from 56.25 to 45.97 MPa (−18.3%), while the average remains essentially unchanged (1.28 vs. 1.29 MPa). The contrast between large changes in peak values and small changes in average values is the key quantitative feature of the comparison: it indicates that the inclusion of intrabiofidelity produces large local stress concentrations and modest changes in the bulk stress state of either tissue.

Principal stresses: The principal stress data refine this picture by separating tensile from compressive contributions. In trabecular bone, maximum principal stress (peak tension) increases from 3.10 MPa in Case 1 to 12.81 MPa in Case 2 (+313%), and minimum principal stress (peak compression, in absolute value) increases from 1.35 to 12.19 MPa (+803%). The trabecular region thus develops both tensile and compressive concentrations of comparable magnitude in Case 2, whereas in Case 1, it was almost entirely in low compression. In cortical bone, maximum principal stress decreases from 57.61 to 46.36 MPa (−19.5%), and minimum principal stress (peak compression) decreases in magnitude from 64.95 to 54.69 MPa (−15.8%). Cortical loading remains predominantly compressive in both cases, with both tensile and compressive peaks reduced by the introduction of intrabiofidelity.

Together, these observations support a coherent physical picture: introducing macro-scale intrabiofidelity in the trabecular region (i) generates local stress concentrations within the trabecular volume that are absent when the trabecular region is represented as a continuous solid, and (ii) reduces the magnitude of stress concentrations in the cortical shell by allowing the trabecular network to participate more actively in load transfer. The bulk stress state of the cortical region is essentially unchanged. The full physical interpretation of this redistribution is developed in Section 4.2.

### 3.4. Volume Control Results (Cases 3A and 3C)

The comparative results of all four cases (Case 1, Case 2, Case 3A, and Case 3C) are reported in the present section. Case 3A (material control with scaled orthotropic properties on continuous geometry) produced a trabecular von Mises peak of 2.749 MPa, only 3.2% above the baseline of Case 1 (2.663 MPa), with a cortical von Mises peak of 63.084 MPa and a total deformation of 0.236 mm. Case 3C (geometric control with spherical cavities and original properties) produced a trabecular von Mises peak of 4.106 MPa, 54.2% above baseline. In stark contrast, the explicit intrabiofidelity model (Case 2) produced a trabecular von Mises peak of 12.102 MPa, 354.5% above baseline (a 4.55-fold increase).

The cortical von Mises peak in Case 3C (67.78 MPa) exceeded those of Cases 1 (56.249 MPa) and 2 (45.972 MPa); inspection of the stress field showed that this peak corresponds to a localized stress concentration at the cortical–trabecular interface adjacent to the spherical cavities closest to the cortical boundary, consistent with the geometric discontinuity introduced by regular spherical porosity. The bulk cortical stress distribution in Case 3C remained predominantly low (<20 MPa across the majority of the cortical surface), comparable to Cases 1 and 2.

Total deformation values for Cases 3A (0.236 mm) and 3C (0.248 mm) were both within the range bounded by Case 1 (0.228 mm) and Case 2 (0.298 mm), consistent with the partial intermediate behavior expected from volume-only controls.

**Figure 12:**
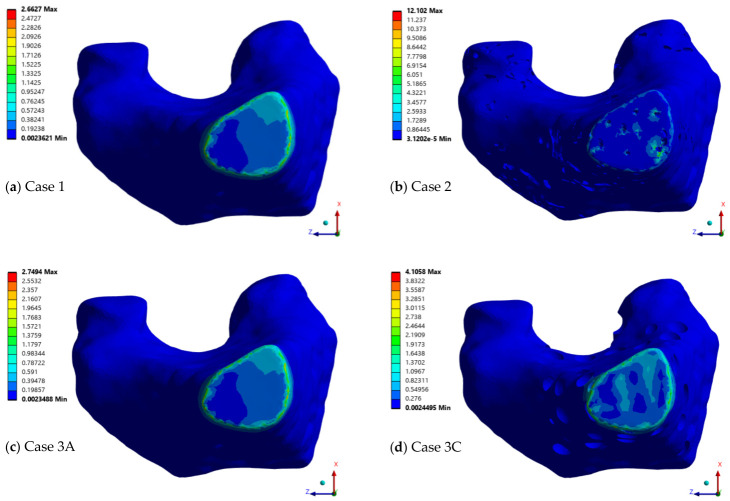
Trabecular von Mises stress distribution in the four comparative finite element models: (**a**) Case 1 (continuous solid, maximum 2.663 MPa); (**b**) Case 2 (intrabiofidelity, maximum 12.102 MPa); (**c**) Case 3A (material control, maximum 2.749 MPa); (**d**) Case 3C (geometric control, maximum 4.106 MPa). Each panel uses its own automatically scaled color range to optimize local contrast; maximum values are reported individually above and should be used for quantitative comparison across cases. Case 2 displays a qualitatively distinct stress distribution relative to the three control cases, supporting the interpretation that the trabecular stress redistribution is driven by the explicit architectural representation rather than by volume reduction alone.

Interpretation: Neither material scaling alone (Case 3A) nor geometric porosity alone (Case 3C) reproduced the magnitude of trabecular stress observed with the explicit intrabiofidelity architecture (Case 2) (Figure 12). Volume reduction, whether modeled through apparent material weakening or through regular geometric cavities, accounts for only a fraction of the redistribution effect. The dominant factor is the explicit biofidel architecture of the trabecular network. This finding supports the methodological premise of intrabiofidelity as a meaningful structural representation, distinct from mere volume reduction.

### 3.5. Morphometric Characterization of the Extracted Trabecular Representation

The extracted trabecular representation used in Case 2 was characterized morphometrically using volumetric measurements of the full trabecular geometry and individual measurements of representative trabecular features (Table 5). The total volume of the trabecular region modeled as a continuous solid (Case 1) was 162,513.09 mm^3^, which serves as the reference enclosing volume (TV) for the trabecular compartment. The volume of the same region after partial extraction of internal porosities (Case 2) was 141,867.12 mm^3^, corresponding to an extracted porosity fraction of 12.7% (1 − BV/TV). The extraction process targeted the largest internal cavities resolvable from the MRI-derived segmentation, but could not be completed exhaustively due to two cumulative constraints: (i) the meshing failures associated with sub-millimetric surface artifacts described in Section 2.7, and (ii) the computational resources available for the extraction iteration on a single-node engineering workstation, as discussed in Section 4.3. The reported value of 12.7% should therefore be interpreted as the fraction of porosity explicitly extracted in the present pipeline implementation, not as the bone volume fraction of the trabecular tissue itself, which is known to lie in the range 0.15–0.30 (i.e., approximately 70–85% porosity) for human distal femoral trabecular bone [42]. The implications of this distinction for the interpretation of the numerical results are discussed in Section 4.2.

Individual trabecular features were further characterized through volume and total surface area measurements of ten representative trabeculae extracted from different regions of the biomodel. From these measurements, an apparent trabecular thickness (Tb.Th-equivalent) was computed for each feature using the standard relation Tb.Th = 2 V/A, where V is the trabecular volume, and A is the total surface area of the feature [42]. The mean apparent thickness obtained across the ten measured trabeculae was 363 ± 108 μm; excluding one outlier feature (12.07 mm^3^, more than four times the volume of the remaining features and likely corresponding to a junction of multiple trabeculae rather than an individual strut), the mean over the remaining nine features was 334 ± 67 μm. Both values exceed the upper bound of the physiological range for human trabecular thickness reported in the literature (100–300 μm; [42]), which is consistent with the resolution limit of the MRI dataset used in this work and with the macro-scale character of the extracted representation. As anticipated in Section 4.3, the present pipeline reproduces the presence of trabecular architecture rather than its native morphometric detail.

Volumetric summary: TV (Case 1, continuous trabecular volume) = 162,513.09 mm^3^; BV (Case 2, after partial porosity extraction) = 141,867.12 mm^3^; extracted porosity fraction = 1 − BV/TV = 0.127 (12.7%).

## 4. Discussion

This work has presented (i) a taxonomic proposal, *intrabiofidelity*, as a complementary descriptor to biofidelity for the explicit documentation of internal tissue architecture in FEA biomodels, and (ii) a pragmatic MRI-based pipeline through which a macro-scale representation of trabecular architecture can be integrated into a distal-femur biomodel using commercial CAD/FEA software. The numerical comparison between Case 1 (external biofidelity only) and Case 2 (with intrabiofidelity) revealed three concurrent patterns: a redistribution of peak stresses between cortical and trabecular compartments, a substantial change in the local stress state of the trabecular region, and a near-invariance of the volume-averaged stress state in either tissue. The interpretation of these results, their scope, and their relationship with the established μFE and hFE literature are discussed below.

### 4.1. Relation to Micro-FE and Homogenized-FE Approaches

The explicit representation of trabecular architecture in FEA is a mature research area. Voxel-based μFE, pioneered by Van Rietbergen and colleagues [13,14] and subsequently developed into a robust methodology through the work of Rüegsegger, Müller, Pahr, Zysset, and others [15,16,17,18,19,20,21,22,23], provides the reference framework for relating trabecular morphometry to apparent and local mechanical behavior, including its application to clinical bone strength prediction [22]. μFE models routinely resolve individual trabeculae at scales of tens of micrometers, use millions to hundreds of millions of elements, and have been extensively validated against experimental mechanical testing of cancellous bone specimens. Homogenized-FE (hFE) approaches, in turn, encode trabecular architecture into continuum-level apparent stiffness and strength tensors, offering a computationally efficient alternative at the cost of giving up explicit geometric representation of the trabecular network.

The present work does not situate itself in either of these categories. The macro-scale trabecular representation extracted from MRI-derived DICOM data and integrated through a CAD solidification step is, by construction, a coarse approximation of the trabecular network. Individual trabeculae are not resolved at their native scale, and the morphometric indices that characterize the real architecture (BV/TV, Tb.Th, Tb.Sp, degree of anisotropy) cannot be expected to coincide with those of μCT-derived models. The contribution of this work is therefore distinct from, and does not compete with, μFE or hFE. It occupies an intermediate region: a biomodel in which the existence of internal porosity is explicit and geometrically represented at a scale compatible with MRI resolution, but in which fine-scale trabecular architecture is not claimed to be reproduced. Within the taxonomy proposed here, such a model has non-zero intrabiofidelity at the macro-scale but low intrabiofidelity at the sub-millimetric scale, and we argue that this distinction is precisely the kind of qualification that the term *intrabiofidelity* is meant to make explicit.

### 4.2. Interpretation of the Numerical Comparison

The comparison between Case 1 and Case 2 under identical orthotropic material assignments and identical boundary conditions revealed three concurrent patterns in the output fields (Table 4): a redistribution of peak stresses between cortical and trabecular compartments, a substantial change in the local stress state of the trabecular region, and a near-invariance of the bulk (volume-averaged) stress state in either tissue. These three observations are consistent with a single underlying physical mechanism, and together they constitute the central quantitative outcome of the present study.

Redistribution of peak stresses: In trabecular bone, peak von Mises stress increases from 2.66 to 12.10 MPa upon introduction of intrabiofidelity, a 4.5-fold elevation. Peak tensile and compressive principal stresses follow the same trend: maximum principal increases from 3.10 to 12.81 MPa (+313%), and minimum principal increases in absolute value from 1.35 to 12.19 MPa (+803%). In cortical bone, peak von Mises stress decreases from 56.25 to 45.97 MPa (−18.3%), maximum principal decreases from 57.61 to 46.36 MPa (−19.5%), and the magnitude of minimum principal decreases from 64.95 to 54.69 MPa (−15.8%). The redistribution is therefore consistent across all three stress measures and reflects the same physical phenomenon viewed from distinct angles.

Change in trabecular stress state: An additional feature of the principal stress data informs the local stress state of the trabecular region. In Case 1 (continuous trabecular), the trabecular bone exhibits very low principal stresses, with minimum principal (peak compression) at only −1.35 MPa and maximum principal (peak tension) at 3.10 MPa: the trabecular volume is essentially in a state of low compression with minor tensile contributions. In Case 2 (with intrabiofidelity), peak compression rises to −12.19 MPa and peak tension to 12.81 MPa, of comparable magnitude. The trabecular region, therefore, transitions from a low-stress, predominantly compressive state to one in which substantial local tensile and compressive concentrations coexist, both of comparable magnitude. The presence of meaningful local tension in Case 2 is mechanically consequential because trabecular bone is known to be more vulnerable to tensile than compressive loading at the tissue level [35,36]; the explicit representation of intrabiofidelity therefore exposes a stress mode that is invisible in a continuous trabecular biomodel.

Near-invariance of the bulk stress state: The bulk stress state, characterized by volume-averaged von Mises stress, is essentially unchanged between cases. In trabecular bone, the average increases from 0.075 to 0.086 MPa (+15%), and in cortical bone it changes from 1.28 to 1.29 MPa (effectively unchanged, +0.5%). The pronounced contrast between large changes in peak values and small changes in average values is the most informative single feature of the present comparison: it indicates that intrabiofidelity primarily affects the *local* stress field through the introduction of stress concentrations at trabecular features, while leaving the global mechanical response of the biomodel essentially intact. This separation of effects (local concentration without bulk perturbation) is generally a desirable property for a methodological pipeline, because it implies that intrabiofidelity provides additional information about local stress fields without compromising the overall mechanical response that would be obtained with a homogenized continuous trabecular representation.

Physical interpretation of the redistribution: The mechanism underlying these observations is straightforward. In Case 1, the trabecular region is represented as a continuous orthotropic solid; the external load applied to the femoral segment is transferred primarily through the cortical shell, which acts as the stiff load-bearing structure due to its order-of-magnitude higher stiffness compared to trabecular bone. The continuous trabecular region, despite its lower apparent stiffness, does not exhibit localized stress concentrations because it lacks geometric features that would concentrate stress. In Case 2, the extracted trabecular representation introduces explicit internal porosity: at the scale of the MRI-derived geometry, the remaining trabecular struts and junctions support concentrated loads at their narrowest cross-sections, producing local peaks of both tension and compression (12.81 and 12.19 MPa respectively) that are absent in Case 1. Concurrently, because the trabecular network is now mechanically engaged in load transfer through its explicit structural connectivity rather than acting as a passive low-stiffness fill, the cortical shell carries a proportionally smaller peak load, and its peak stresses are reduced by approximately 16–20%. This behavior is consistent with the classical observation in bone biomechanics that the cortical and trabecular compartments share load through the cortical–trabecular interface, with the proportion carried by each compartment depending on the effective stiffness and connectivity of the trabecular network [19,20,23].

Asymmetry in displacement fields: The displacement fields exhibit a related, secondary feature. In Case 1, peak trabecular displacement (0.228 mm) is approximately 9% higher than peak cortical displacement (0.209 mm), reflecting the difference in apparent stiffness between the two tissues. In Case 2, this gap widens substantially: peak trabecular displacement (0.298 mm) is 46% higher than peak cortical displacement (0.204 mm). The peak displacements occur in spatially close but non-coincident regions of the biomodel: the cortical maximum is located on the inner border of the proximal diaphyseal cut where the load is applied, while the trabecular maximum is located on a trabecular feature of the immediately adjacent superior region. The bonded contact between cortical and trabecular surfaces (Section 2.4, Table 1) ensures continuity of displacement at the interface but does not enforce equality of peak displacements throughout the volumes. The widened gap in Case 2 reflects increased local compliance of the trabecular network at points where porosity reduces the effective load-bearing cross-section, while the cortical shell, partially relieved through the load redistribution described above, exhibits slightly lower peak displacements than in Case 1. The fact that the peak locations remain in the same anatomical region across cases supports the interpretation that the redistribution observed here is a local mechanical effect of intrabiofidelity rather than an artifact of altered global load paths.

Three cautions apply to the interpretation of these results. First, as detailed in Section 2.7, the Case 2 mesh could not be refined further due to sub-millimetric geometric artifacts in the extracted trabecular representation, so a conventional h-refinement convergence study was not completed; the magnitude of the stress redistribution observed here must therefore be interpreted within the discretization limitations of the current pipeline. Second, peak von Mises and principal stress values in the trabecular region of Case 2 are confined to very small volumes, and their specific magnitudes are sensitive to the local mesh topology around the narrowest trabecular features; the *direction* of the stress redistribution (increase in trabecular peaks, decrease in cortical peaks, near-invariance of bulk averages) is a robust qualitative observation, while the specific magnitudes should be reported as indicative rather than quantitatively predictive. Third, the trabecular geometry reproduced at MRI resolution is coarser than the native trabecular architecture, and finer-scale representations (from μCT data) would in principle refine the numerical magnitudes while preserving the qualitative load-redistribution pattern. These limitations do not compromise the methodological contribution of the pipeline, but they delimit the scope of the quantitative claims that the present results can support.

An additional consideration concerns the magnitude of porosity actually represented in Case 2. As reported in Section 3.5, the extracted porosity fraction in the present pipeline implementation was 12.7%, well below the physiological porosity range of human trabecular bone (typically 70–85%, corresponding to BV/TV ≈ 0.15–0.30). The Case 2 biomodel, therefore, substantially under-represents the volumetric porosity of real trabecular tissue, while still introducing geometric discontinuities sufficient to produce the load redistribution reported in Table 4 and Table 5. The observed redistribution, a 4.5-fold increase in peak trabecular von Mises stress and an 18.3% reduction in peak cortical von Mises stress relative to the continuous case, therefore, corresponds to the effect of partial intrabiofidelity, and should be interpreted as a conservative lower bound on the redistribution that would be obtained with a more thoroughly extracted (or μCT-resolved) trabecular geometry. The fact that even partial macro-scale extraction produces a quantitatively significant effect supports the relevance of the intrabiofidelity framework for applications in which local stress fields are of interest, particularly orthopedic device design.

### 4.3. Limitations

Constitutive assumptions of orthotropic property assignment: The orthotropic constants applied to the trabecular compartment in both Cases 1 and 2 (Table 2) represent tissue-level elastic properties of bone matrix, independent of macroscopic architecture. In Case 1 (continuous solid), these properties serve as apparent-level surrogates that account collectively for both intrinsic tissue anisotropy and unresolved architectural effects, a standard assumption in macro-scale FE models of bone (Pahr and Zysset [20,21]; Zysset et al. [22]). In Case 2 (intrabiofidelity), the same constants are applied at the tissue level to each geometrically resolved trabecular element, methodologically consistent with µFE approaches (van Rietbergen et al. [13,14]). The apparent-level constants in Case 1 and the tissue-level constants in Case 2 are not strictly identical material descriptors; this is a known limitation of multi-scale FE modeling of bone and warrants future formal calibration against µFE reference data.

Segmentation artifacts and local stress concentrations: The biomodel reconstruction process from MRI data inherently introduces residual geometric irregularities along trabecular edges, particularly at concavities and branching points. While ScanIP (Simpleware) surface smoothing reduces the staircase effect typical of voxel-based segmentation, some residual irregularities act as numerical stress raisers that may locally overestimate true physiological peak stresses. Consequently, the trabecular von Mises peak values reported in Case 2 (e.g., 12.102 MPa) should be interpreted as upper-bound estimates rather than exact physiological magnitudes. The qualitative redistribution effect (cortical reduction, trabecular increase) is robust to this limitation, as it manifests at the bulk distribution level and is symmetric across the cortical and trabecular compartments under the same meshing strategy.

Mesh quality and convergence study: A formal multi-density mesh convergence study on Case 2 was attempted but precluded by the geometric complexity of the intrabiofidelity trabecular reconstruction. Attempts to refine the element size repeatedly produced patch-conforming mesher failures associated with sharp or extremely thin trabecular features and high-curvature regions, generating errors such as “surface mesh is intersecting or close to intersecting” and “patch-conforming tetrahedron mesh failed because of an edge intersection in the boundary mesh”. These failures persisted across multiple meshing strategies and could consume several days of iterative work per attempt without converging to a stable mesh, which made a systematic multi-density study impractical within the timeframe of the present study. The reference mesh, therefore, represents the densest stable configuration achievable with the current geometry, and any further refinement is conditional upon successful resolution of these meshing constraints. The reference mesh (1,831,736 elements; 2,987,600 nodes; mean element quality = 0.6835, standard deviation = 0.171; element quality range 3.04 × 10^−3^ to 0.9998) represents the densest stable configuration achievable. Mesh independence is supported indirectly by (i) the mean element quality value, which exceeds the ANSYS threshold of 0.5 for biomedical applications; (ii) the consistency of bulk-average von Mises values within 1% across compartments; and (iii) qualitative agreement with published µFE results for trabecular bone (van Rietbergen [13,14]; Nawathe et al. [43]). The small fraction of degenerate elements (maximum skewness approaching 1.0) is concentrated at thin trabecular junctions; their effect is localized and does not corrupt the bulk solution, as confirmed by the absence of solver warnings and the physical consistency of the global stress redistribution. Formal mesh convergence in geometrically complex intrabiofidelity models is identified as a methodological priority for future work, potentially requiring alternative meshers (e.g., hexahedral-dominant or polyhedral) or geometric simplification strategies.

Single-specimen and single-loading-condition design: The present study employs a single biomodel reconstruction and a single-loading condition (axial compression, 0.5517 MPa over 800.17 mm^2^). The findings are therefore positioned as a methodological proof-of-concept rather than a population-level or clinically generalizable quantitative result. This framing is consistent with foundational studies that established the µFE approach on single specimens prior to multi-specimen validation (van Rietbergen et al. [13,14]; Müller and Rüegsegger [15]). Multi-specimen, multi-loading studies are necessary to establish quantitative thresholds and population-level intrabiofidelity benchmarks.

Loading condition simplification: The applied axial compressive loading is a substantial simplification relative to in vivo conditions, where the distal femur is subjected to multiaxial forces from quadriceps and hamstring musculature, patellar tendon tension, and collateral ligament constraints. These would generate bending, torsion, and shear components in addition to axial compression, potentially modifying both the spatial distribution and the magnitude of stress redistribution between Cases 1 and 2. The choice of simplified axial loading in the present work is justified by its methodological purpose: to isolate the effect of architectural representation under reproducible, controlled loading. Extension to physiological multiaxial loading is identified as a priority for subsequent work.

MRI resolution and morphometric ceiling: The MRI acquisition used in this study resolves trabecular features with a mean thickness on the order of 363 µm (Table 5). Trabecular features below the imaging resolution limit are not captured by the reconstruction and are subsumed implicitly into the apparent-level material properties assigned to the bulk compartment. This represents a fundamental ceiling on the achievable level of intrabiofidelity using clinical MRI. Higher-resolution modalities (HR-pQCT, µCT) would capture finer trabecular features and are expected to amplify the stress redistribution effects observed here. The intrabiofidelity classification framework proposed in Section 2.9 is intended to accommodate this resolution-dependent gradient.

Computational resources: All finite element analyses were conducted on a workstation with an AMD Ryzen 7 4800H processor, 32 GB DDR4 RAM (3200 MT/s), and an NVIDIA GeForce RTX 2060 GPU (6 GB dedicated memory), using ANSYS Workbench 2025 R2 with the direct sparse solver. Approximate solution times were 2 h for Case 2 (most computationally demanding, 1.83 million elements) and 30–45 min each for Cases 1, 3A, and 3C.

Several limitations of the present study must be explicitly acknowledged.

**Mesh refinement and convergence:** A conventional h-refinement convergence study for Case 2 could not be completed with the current biomodel. As detailed in Section 2.7, the macro-scale trabecular geometry extracted from MRI-derived DICOM data contains sub-millimetric surface artifacts (effective edge lengths of the order of 10^−5^ mm) that generate degenerate elements at the upper tail of the skewness distribution. Mitigation through adaptive Defeature Size and automatic Virtual Topology (low and medium behaviors) did not eliminate these features without simultaneously removing anatomically meaningful trabecular detail, and further refinement of the global element size produced meshing failures. Additionally, mesh refinement beyond the reported density was constrained by the available computational infrastructure (a standard engineering workstation, single-node configuration). μFE-class refinements of comparable trabecular geometries typically require parallel high-performance computing resources, which were not available in the present study; this constraint, rather than a methodological choice, dictated the upper bound of mesh density reported in Section 2.7. The accessibility of the proposed pipeline on standard workstations, however, is a deliberate feature of the framework, intended for groups operating outside of HPC-equipped research centers (Section 4.4). The reported mesh, therefore, represents the highest-quality discretization attainable for the present trabecular representation within standard ANSYS Workbench meshing on workstation-class hardware.

**Imaging resolution:** The trabecular representation was extracted from MRI-derived DICOM data. MRI does not resolve individual trabeculae at their native scale (typical trabecular thickness of 100–300 μm), which places a hard upper bound on the achievable intrabiofidelity of the resulting biomodel. The macro-scale trabecular structure presented here should therefore be understood as a representation of the *presence* of internal porosity, not as a morphometrically accurate reproduction of the trabecular network. Morphometric indices of the extracted representation are reported in Section 3.5 to document this limitation quantitatively; extension of the pipeline to CT datasets with higher spatial resolution, or to μCT where available, is a natural direction for future work.

**Validation:** The present version of the study compares two internally consistent biomodels but does not include experimental validation or direct comparison with published μFE or experimental measurements of distal-femur mechanics. This is a substantial limitation for any quantitative claim about predictive accuracy, and the results of the Case 1 vs. Case 2 comparison should therefore be interpreted as illustrating the *effect* of including intrabiofidelity within a given modeling framework, not as a validated prediction of bone behavior. Comparative validation against experimental displacement or stiffness data for the distal femur, and against μFE predictions for equivalent loading, are the next steps before the pipeline can be applied in predictive contexts.

Loading conditions: The external load of 441.45 N applied to the knee corresponds to a bipodal-standing approximation based on body-weight distribution and does not include muscular contributions to tibiofemoral contact force. Physiological in vivo measurements with instrumented knee implants [31,33] have consistently shown that tibiofemoral contact forces during quiet standing and walking substantially exceed body-weight-only estimates due to muscle co-contraction; comparable patient-specific FE studies including muscle and ligamentous boundary conditions have produced contact-force estimates several times higher than the body-weight-only approximation [32]. The loading scenario used here was selected for didactic clarity and for direct comparability between Case 1 and Case 2; it is not intended as a physiological prediction.

Stress concentrations at the diaphyseal cut: Peak von Mises and principal stresses in both Case 1 and Case 2 are localized in the proximal region of the biomodel, on the inner border of the diaphyseal cut where the external load is applied. This localization reflects the well-known geometric concentration of stress at the boundary of a free edge under applied traction, and is therefore an artifact of the boundary-condition geometry rather than a physiological feature of the distal femur under bipodal standing. The redistribution observed between Case 1 and Case 2 (Section 4.2) and, in particular, the qualitative change in trabecular stress state from predominantly compressive to mixed tensile-compressive is interpreted relative to this common geometric reference: both cases share the same external geometry and boundary conditions, so the differences between them are attributable to the inclusion of intrabiofidelity rather than to the cut-edge concentration itself. The numerical magnitudes of peak stresses, however, should be interpreted within this geometric context, and direct comparison with anatomically intact biomodels (where the femur is loaded through the hip joint rather than through a transverse cut) would refine the absolute values.

Model scope: The biomodel includes the distal femur, both menisci and their contacts with the cortical bone of the femur. The tibia, articular cartilage, and main ligamentous structures of the knee are not represented. This reduction is consistent with the methodological focus of the study and has no bearing on the demonstration of the intrabiofidelity pipeline, but it does restrict any clinical or biomechanical interpretation of the results.

Manual extraction workflow: The extraction of the trabecular representation was performed through a manual, slice-by-slice procedure within ScanIP^®^. This procedure is reproducible and does not require specialized software beyond the commercial tools listed, but it is labor-intensive and operator-dependent. Automation of the extraction step is a concrete direction for methodological refinement.

### 4.4. Implications and Use Cases

Adopting intrabiofidelity as an explicit descriptor provides a compact way of stating, in reporting FEA biomodels, which internal attributes of the tissue have been reproduced and at what scale. For the literature reviews and meta-analyses of FEA studies in orthopedics and applied biomechanics, this vocabulary allows reviewers to classify existing work along the external-vs-internal and scale axes in a systematic way, and to identify gaps in the spectrum between fully continuous biomodels and full μFE models. For didactic purposes, the distinction between biofidelity and intrabiofidelity clarifies to students of biomechanics and bioengineering what fidelity actually refers to in the modeling of tissues whose mechanical behavior depends on internal architecture.

The pragmatic pipeline described here is not proposed as a substitute for μFE in research contexts where μCT data and high-performance computing are available; μFE remains the gold standard for quantitative prediction of trabecular bone mechanics [13,14,15,16,17,18,19,20,21,22,23]. The pipeline is operable on standard engineering workstations, without requiring parallel HPC infrastructure or μCT data; this accessibility is essential for the intended use cases listed below. It is proposed as a feasible alternative for three specific use cases. First, consider research contexts in which μCT data are unavailable, and MRI is the primary imaging modality, which is a common situation in clinical musculoskeletal biomechanics, in patient-specific studies, and in research groups operating outside of specialized μCT facilities. Second, teaching and training contexts in biomechanical FEA, in which the didactic value of working with a biomodel that explicitly incorporates internal architecture outweighs the need for quantitative accuracy against μFE. Third, preliminary design of orthopedic elements (screws, plates, nails, prostheses) in which stress concentration effects associated with the presence, rather than the precise geometry, of internal porosity need to be considered [10]; in such contexts, a macro-scale intrabiofidel biomodel can reveal qualitative effects that a fully continuous biomodel cannot show, while remaining computationally tractable on standard workstations.

### 4.5. Comparison with Published Data

Quantitative benchmarking with published data: The cortical von Mises peak reduction observed between Cases 1 and 2 (−18.3%) is qualitatively consistent with the cortical–trabecular load-sharing findings of Nawathe et al. [43], who reported that trabecular bone carries approximately 30–65% of total load in the femoral neck depending on loading configuration. Implicit in their result is a corresponding reduction in apparent cortical load share when trabecular contribution is properly modeled, a redistribution that continuum models systematically underestimate.

The 4.55-fold increase in trabecular peak von Mises (from 2.663 MPa in Case 1 to 12.102 MPa in Case 2) is in directional agreement with Jia et al. [44], who demonstrated experimentally that removal of cancellous bone reduced femoral failure load by 67.2%, implying that intact trabecular architecture carries substantial mechanical load that is not captured by purely continuum representations. Direct quantitative comparison across studies is constrained by differences in loading magnitude, anatomical region, and boundary conditions.

The trabecular von Mises range observed in Case 2 (~0.005–12.1 MPa, with bulk mean values on the order of 3–4 MPa across the compartment) lies broadly within the range reported in µFE studies of trabecular bone subjected to comparable loading (van Rietbergen et al. [13,14]: ~1–15 MPa; Bevill et al. [18]: ~2–20 MPa). The agreement at the order-of-magnitude level provides indirect support for the physical plausibility of the intrabiofidelity stress predictions, while exact quantitative agreement is not expected given differences in resolution and loading regime.

Although the present study does not include direct experimental validation, the numerical values obtained for Case 1 and Case 2 can be situated within the range of published FEA and μFE results for the human femur under comparable loading conditions. This comparison is presented as an order-of-magnitude consistency check, not as a quantitative validation.

Peak von Mises stresses in trabecular bone: Liu et al. [45] reported a maximum von Mises stress of 24.04 MPa in cancellous bone for a one-legged stance loading condition (L1) in a proximal-femur FEA model. The trabecular peak of 12.10 MPa obtained in Case 2 of the present study is approximately half of this value, of the same order of magnitude, and within the range expected for cancellous bone in a femoral biomodel under a bipodal-standing load. In the lumbar vertebral body, Rohlmann et al. [46] reported median peak von Mises stresses in cancellous bone in the range 1.5–4.5 MPa across standing and several other loading conditions. Our Case 1 trabecular peak (2.66 MPa) falls within this range, and the volume-averaged von Mises across the trabecular volume in Case 1 (0.075 MPa) is consistent with a low-stress bulk state of cancellous tissue under standing conditions.

Cortical peak stresses: The cortical peak von Mises stresses obtained in the present study (56.25 MPa in Case 1 and 45.97 MPa in Case 2) fall within the range typically reported for cortical bone in distal-femur FEA studies under standing-type loading. These values are well below the cortical yield strength of 115 MPa (Table 2), consistent with the elastic regime under the imposed bipodal load.

Cortical–trabecular load redistribution: The mechanism underlying the redistribution observed in our results is consistent with the load-sharing patterns documented at finer scales by Nawathe et al. [43]. Using μCT-based μFE with approximately 280 million elements per model on 18 human proximal femora, those authors quantified the fraction of bending moment carried by cortical versus trabecular bone in the femoral neck under stance loading and found that the cortical bone takes 88 ± 5% of the load distally, while the trabecular bone carries the majority of the load proximally. The redistribution observed in the present study is qualitatively consistent with this picture, demonstrating at the macro-scale a behavior whose existence and quantitative significance had been demonstrated previously at sub-millimetric resolution.

Magnitude of the trabecular contribution: An independent test of the importance of representing trabecular bone explicitly is provided by Jia et al. [44], who used finite element analysis on 20 geometric models (10 intact, 10 cancellous-excised) of the proximal femur and reported that removal of cancellous bone reduced femoral failure load by 67.2% on average. Although that study used different outputs and loading conditions, the result establishes the same qualitative point that motivates the present work: the trabecular compartment carries a substantial portion of the mechanical role of bone, and biomodels that omit or homogenize trabecular architecture systematically underestimate the trabecular contribution to local stress fields.

Limitations of this comparison: Three caveats apply to the comparison presented above. First, the cited studies use different anatomical sites (proximal femur, vertebral body) than the present analysis (distal femur). Second, the loading conditions and magnitudes differ across studies. Third, the cited μFE studies use μCT-derived geometry at sub-millimetric resolution, whereas our pipeline uses MRI-derived geometry at the macro-scale. The combined picture nonetheless supports the interpretation that the values reported in Section 3 and Section 4.2 are within the range of what the broader μFE and FEA literature considers physically reasonable for the human femur under static or quasi-static loading.

## 5. Conclusions

This work has introduced *intrabiofidelity* as a taxonomic descriptor, complementary to biofidelity, for the explicit characterization of internal tissue architecture in finite element biomodels. The term is not proposed to claim novelty in the physical representation of trabecular architecture (a mature problem addressed by micro-finite element analysis since the mid-1990s) but to provide a systematic vocabulary that makes the external-vs-internal distinction explicit in how biomodels are documented, compared, and discussed. A pragmatic MRI-based pipeline has also been described through which a macro-scale representation of trabecular architecture can be integrated into FEA biomodels using commercial CAD/FEA software, without requiring micro-CT imaging or high-performance computing resources.

A numerical comparison was performed between a biomodel with external biofidelity only (Case 1) and an equivalent biomodel incorporating macro-scale intrabiofidelity in the trabecular bone (Case 2), under identical orthotropic material assignments, boundary conditions, and loading. Three concurrent patterns characterized the comparison. First, peak stresses redistributed between compartments: trabecular peak von Mises stress increased from 2.66 to 12.10 MPa (a 4.5-fold elevation), while cortical peak von Mises stress decreased from 56.25 to 45.97 MPa (an 18.3% reduction); the same pattern held for principal stresses. Second, the local stress state of the trabecular region transitioned from a low-stress, predominantly compressive state in Case 1 to one in which substantial local tensile and compressive concentrations of comparable magnitude coexist in Case 2. Third, the bulk stress state of either tissue, characterized by volume-averaged von Mises stress, remained essentially unchanged: trabecular average changed from 0.075 to 0.086 MPa, and cortical average from 1.28 to 1.29 MPa. The pronounced contrast between large changes in peak values and small changes in averages indicates that intrabiofidelity primarily affects local stress concentrations rather than the bulk mechanical response.

Methodological limitations are explicitly acknowledged. The macro-scale trabecular geometry extracted from MRI-derived DICOM data contains sub-millimetric surface artifacts that prevented a conventional h-refinement convergence study with the current pipeline, and geometric clean-up of these artifacts is identified as a priority for future work. MRI resolution places a hard upper bound on the morphometric accuracy of the extracted trabecular representation. Experimental validation, direct comparison with micro-FE references, extension to physiologically realistic loading conditions, and application to the preliminary design of orthopedic devices are identified as priority directions for future work.

The contribution of this work is methodological and terminological. *Intrabiofidelity* is proposed as a descriptor that makes explicit an attribute of biomodels that has typically been left implicit in the applied FEA literature, and the pipeline described here offers a feasible route for incorporating macro-scale internal architecture into biomodels using routinely available imaging and commercial software. The framework complements, rather than competes with, the established micro-FE and the homogenized-FE literature on trabecular bone mechanics.

## Figures and Tables

**Table 1:** Contact definitions used in the finite element model.

Interface	Contact Type	Behavior
Femoral cortical bone ↔ Femoral trabecular bone	Bonded	Tissue continuity
Femoral cortical bone ↔ External meniscus	Frictionless	Articular contact
Femoral cortical bone ↔ Internal meniscus	Frictionless	Articular contact
Femoral trabecular bone ↔ Trabecular representation	Bonded	Intrabiofidelity integration

**Table 2:** Mechanical properties of tissues used in the finite element analysis [6,25,34,38].

Material	Property	*X*-Axis	*Y*-Axis	*Z*-Axis
Cortical bone [6,25]	Young’s modulus, E (MPa)	9600	9600	17,800
	Shear modulus, G (MPa)	3100	3510	3510
	Poisson’s ratio, ν	0.55	0.30	0.30
Trabecular bone [6,25]	Young’s modulus, E (MPa)	144.0	99.0	344.0
	Shear modulus, G (MPa)	53.0	63.0	45.0
	Poisson’s ratio, ν	0.23	0.11	0.13
Meniscus [34]	Young’s modulus, E (MPa)	55		
	Shear modulus, G (MPa)	18.97		
	Poisson’s ratio, ν	0.45		
	Density (g/cm^3^)	1.10		

Notes. E = Young’s modulus along principal orthotropic axes; G = shear modulus; and ν = Poisson’s ratios. The orthotropic *Z*-axis is aligned with the long axis of the femur. Meniscus values correspond to isotropic linear-elastic assumptions consistent with static loading [38].

**Table 3:** Mesh statistics for Case 1 (without intrabiofidelity) and Case 2 (with intrabiofidelity).

Parameter	Case 1 (Without Intrabiofidelity)	Case 2 (With Intrabiofidelity)
Element type	SOLID187 (Tet10)	SOLID187 (Tet10)
Number of nodes	610,883	2,987,600
Number of elements	355,461	1,831,736
Skewness, average	0.428	0.450
Skewness, maximum	0.99987	1.000
Bounding box diagonal (mm)	194.29	194.29

Note: Skewness metric: average values in the range 0.25–0.50 correspond to “good” mesh quality in ANSYS Workbench conventions; values > 0.95 denote degenerate elements. The maximum skewness of 1.000 in Case 2 corresponds to isolated degenerate elements at sub-millimetric features of the extracted trabecular geometry (see main text).

**Table 4:** Summary of peak values of total deformation, von Mises stress (maximum and average), and principal stresses (maximum tensile and maximum compressive) for trabecular and cortical bone. Comparison between Case 1 (without intrabiofidelity) and Case 2 (with intrabiofidelity) under orthotropic material assignments.

Output Variable	Tissue	Metric	Case 1	Case 2
Total deformation (mm)	Trabecular	Maximum	0.22817	0.29782
	Cortical	Maximum	0.20945	0.20443
Von Mises stress (MPa)	Trabecular	Maximum	2.663	12.102
	Trabecular	Average	0.0746	0.0858
	Cortical	Maximum	56.249	45.972
	Cortical	Average	1.283	1.293
Maximum principal stress (MPa)	Trabecular	Maximum	3.101	12.805
	Cortical	Maximum	57.607	46.355
Minimum principal stress (MPa)	Trabecular	Minimum	−1.346	−12.185
	Cortical	Minimum	−64.953	−54.690

Note: Both cases share identical material properties, contacts, boundary conditions, and external loading (Section 2), with orthotropic material properties assigned to both cortical and trabecular bone (Table 2). Case 1 (without intrabiofidelity) was discretized using 355,461 SOLID187 elements and 610,883 nodes; Case 2 (with intrabiofidelity) uses 1,831,736 elements and 2,987,600 nodes (Table 3). The ~5× difference in element count reflects the additional geometric detail introduced by the extracted trabecular representation in Case 2. Maximum principal stress values are positive and represent the magnitude of the largest local tension; minimum principal stress values are negative and represent the magnitude of the largest local compression (in absolute value). All peak values correspond to localized concentrations within the volume; average von Mises values are reported separately to characterize the bulk stress state of each tissue.

**Table 5:** Morphometric characterization of the extracted trabecular representation. Volumes and surface areas of ten individual trabecular features measured in ANSYS^®^ SpaceClaim, with apparent trabecular thickness computed as Tb.Th = 2V/A.

Trabecula	Volume (mm^3^)	Total Surface Area (mm^2^)	Apparent Tb.Th (mm)
1 *	12.0699	38.9294	0.620
2	0.6377	4.8726	0.262
3	1.3289	7.5204	0.353
4	0.7744	4.7733	0.324
5	1.9429	8.5512	0.454
6	2.8731	15.7276	0.365
7	0.3571	2.816	0.254
8	0.6898	4.5132	0.306
9	0.6283	4.3559	0.288
10	1.344	6.6619	0.403
Mean ± SD (*n* = 10)	—	—	0.363 ± 0.108
Mean ± SD (*n* = 9) †	—	—	0.334 ± 0.067

Notes. * Trabecula 1 is treated as an outlier: its volume (12.07 mm^3^) is more than four times that of the remaining nine features and most likely corresponds to a junction or convergence zone of multiple trabeculae rather than an individual trabecular strut. † Mean and SD computed excluding Trabecula 1. Reference physiological ranges for human trabecular bone: BV/TV ≈ 0.15–0.30; Tb.Th ≈ 100–300 μm [42].

## Data Availability

The data supporting the findings of this study are available from the corresponding author upon reasonable request.
